# A Rapid Point-of-Care Ultrasound Diagnosis and Treatment of Tamponade in a Patient With Rare and Lethal Purulent Pericarditis: A Case Report

**DOI:** 10.7759/cureus.77205

**Published:** 2025-01-09

**Authors:** Autumn N Bass, Sean Lynch, Charlotte Derr, James Gillen

**Affiliations:** 1 Emergency Medicine, University of South Florida Morsani College of Medicine, Tampa, USA

**Keywords:** echocardiography - pericardial - effusion, pericardial effusion. cardiac tamponade, pocus in emergency medicine, pocus (point of care ultrasound), pulsus paradoxus

## Abstract

This article describes the prompt diagnosis and life-saving intervention facilitated by point-of-care ultrasound (POCUS) in a patient presenting with tamponade secondary to a rare case of purulent pericarditis. Within a remarkable timeframe of only 40 minutes from emergency department (ED) presentation to pericardial drainage, POCUS not only confirmed the diagnosis but also expedited critical treatment measures. The etiology of the underlying purulent pericardial effusion was traced to a *Streptococcus pneumoniae* infection. Ultimately, the patient had untreated X-linked agammaglobulinemia (XLA), a rare immunodeficiency disorder, adding layers of complexity to the clinical scenario. Notably, only three other documented cases exist in the literature detailing adult XLA patients afflicted with purulent pericarditis, rendering this case both exceptional and informative.

## Introduction

Point-of-care ultrasound (POCUS) is a critical skill required for all residents enrolled in emergency medicine training programs. It can quickly identify life-threatening conditions that traditionally demanded prolonged diagnostic timelines spanning hours to days. Integration of POCUS in the emergency department (ED) facilitates earlier intervention and treatment, yielding improved outcomes. Door-to-needle time (DTNT) is a parameter emphasized and measured in both the cardiology and stroke neurology literature. In this article, we report a rare case of cardiac tamponade caused by purulent pericarditis in an adult patient with agammaglobulinemia. The diagnosis was made immediately with bedside ultrasound with resultant pericardial puncture and drainage in the cath lab within a mere 40 minutes of the patient’s arrival to the ED. Given the high mortality associated with purulent pericarditis, quick recognition and intervention were paramount. The uniqueness of this case lies in several facets: firstly, the rarity of agammaglobulinemia manifesting in an adult patient; secondly, the unprecedented efficiency with which life-saving measures were enacted; and thirdly, purulent pericarditis, a seldom-seen complication of agammaglobulinemia further underscores the atypical nature of this presentation.

## Case presentation

**Figure 1:**
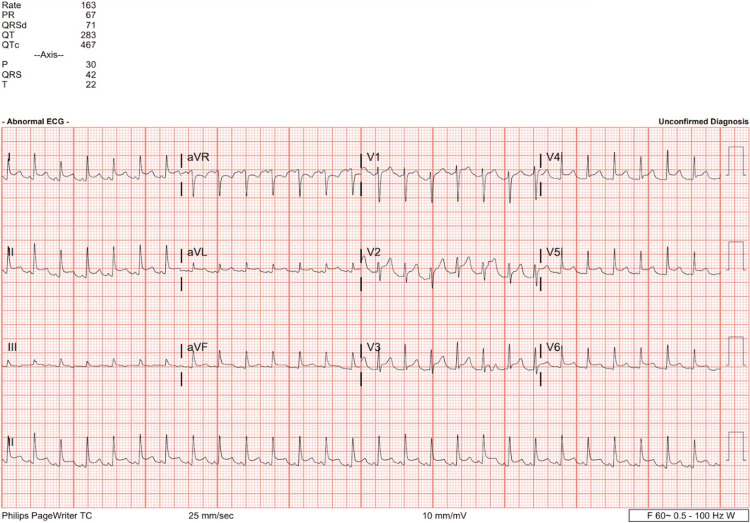
Initial electrocardiogram (EKG) interpreted as sinus tachycardia with ST-segment elevations in multiple leads (leads I, II, aVL, V2, V3, V4, V5, and V6) and ST-segment depression in aVR consistent with pericarditis

A 26-year-old male presented to the ED with a two-day history of chest pain. Upon initial evaluation, the patient was noticeably uncomfortable due to his chest pain. He was noted to be profusely diaphoretic and mildly dyspneic. The patient stated that about three days before the presentation, he had developed a cough, fevers, and general malaise, with chest pain developing on the day after the onset of symptoms. The patient indicated that the severity of the chest pain had progressed, prompting him to seek evaluation in the ED. The patient presented with the following initial vital signs: heart rate of 160 bpm, blood pressure of 106/64, respiratory rate of 38, and temperature of 96.8°F. The patient's reported past medical history included asthma and a previous history of pneumonia. Upon arrival at the ED, the patient was triaged by an emergency physician who obtained an EKG and promptly moved him to a room for continued management. The EKG showed diffuse ST segment elevations in leads I, II, aVL, V2, V3, V4, V5, and V6, with depressions only in augmented vector right (aVR), prompting suspicion of pericarditis or myocarditis (Figure 1).

**Figure 2:**
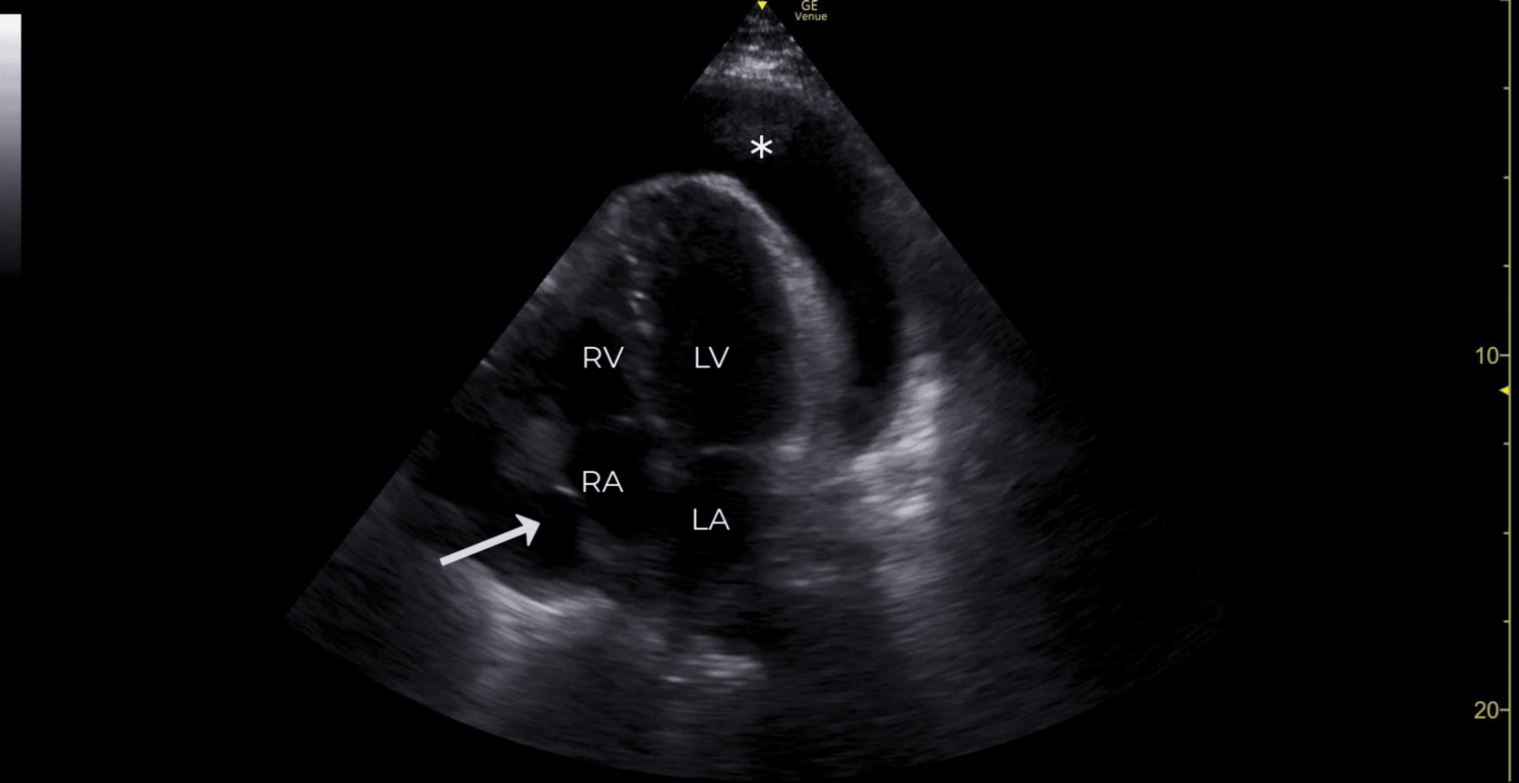
Apical four-chamber view with large pericardial effusion (*) causing systolic collapse (arrow) of the right atrial (RA) wall consistent with tamponade

**Figure 3:**
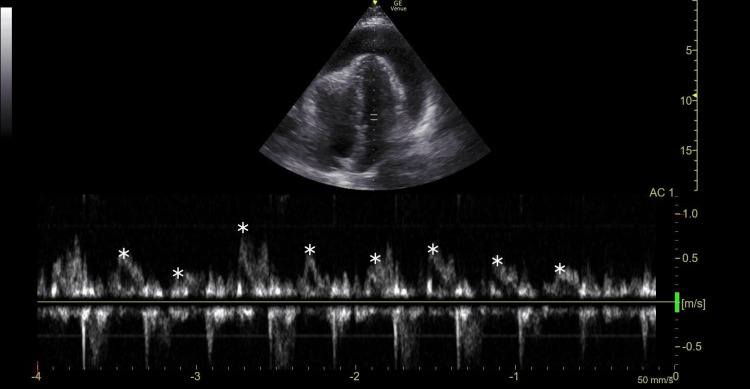
Apical four-chamber view with pulsed wave Doppler imaging demonstrating respiratory-phasic velocity changes of the mitral valve inflow velocities (*)

The patient was started on broad-spectrum antibiotics and crystalloid fluids as part of the ED’s sepsis-alert protocol. Due to the ill appearance of the patient and the concern for an underlying cardiac source for symptoms, an emergent POCUS was performed. The ECHO revealed a large pericardial effusion (Figure 2, Video 1) with systolic collapse of the right atrium (RA) and diastolic collapse of the right ventricle (RV). The mitral valve and tricuspid valve in-flow velocities (Figure 3) demonstrated inspiratory and expiratory variations greater than 25% and 40%, respectively, suggestive of tamponade physiology. Cardiology was consulted, and the patient was urgently taken to the cath lab to place a pericardial drain. The time from ED arrival to pericardial puncture (“DTNT”) was 40 minutes.

**Video 1:** Apical four-chamber view with notable pericardial effusion

While the patient was receiving treatment in the cath lab, the ED lab results returned with a white blood cell (WBC) count of 43.84 (95% neutrophils and <2% lymphocytes), creatine phosphokinase (CPK) of 39, erythrocyte sedimentation rate (ESR) of 112, procalcitonin of 11.09, ferritin of 1,292.8, and a negative COVID test result. A viral panel was positive for rhinovirus. A chest X-ray showed a moderate-sized right pleural effusion with concern for overlying infiltrates. In the cath lab, more than 500 cc of hazy, straw-like pericardial fluid was removed and sent for various cytologic analyses as part of a broad infectious source evaluation. The resulting cytology demonstrated >25 WBCs per 100× field, suggesting purulent pericarditis. After the placement of the pericardial drain, the patient's heart rate decreased to 80 bpm; however, his post-pericardiocentesis comprehensive echocardiogram demonstrated a severely reduced ejection fraction of 30% to 35%, and therefore, there was ongoing concern for possible underlying myocardial involvement. Following the pericardiocentesis, the patient was admitted to the cardiac care unit (CCU) for continued management. A secondary medical history was obtained after arrival at the CCU, revealing that the patient had been diagnosed with X-linked agammaglobulinemia (XLA) at the age of 19. He had been under the care of an immunology outpatient clinic and was previously receiving immunoglobulin replacement therapy every two weeks. However, he had stopped this treatment three years earlier after being lost to follow-up. The inpatient cardiology service consulted Immunology, who reevaluated the patient's immunoglobulin levels (IgG: <109, IgA: <5, IgM: <5) and recommended continued broad-spectrum antibiotic coverage along with the administration of intravenous immunoglobulin. The patient's flow cytometry suggested CD19+ B-cells but negligible CD18 levels, indicating the impaired function of the Bruton tyrosine kinase (BTK) signal cascade. Cultures from the pericardial fluid grew *Streptococcus pneumoniae* species, and antibiotics were later adjusted for targeted treatment. A chest CT confirmed parapneumonic effusions with loculations felt to be a consequence of bilateral multifocal pneumonia, leading to the placement of a left-sided chest tube for fibrinolytic therapy. A cardiac MRI was performed due to the persistent reduction of his ejection fraction post-pericardiocentesis and showed diffuse pericardial thickening with ventricular impedance, providing evidence of constrictive pericarditis and contributing to the reduced ejection fraction. While hospitalized, the patient exhibited progressive improvement, marked by the resolution of clinical symptoms, subsequent removal of the patient’s chest tube, normalization of hemodynamics, and a decline in infectious markers. This positive progress culminated in the patient's discharge to home, accompanied by ongoing immunoglobulin replacement therapy and close follow-up with immunology and cardiology specialists.

## Discussion

Agammaglobulinemia is an inherited immunodeficiency disorder characterized by low or absent B cells, leading to underproduction or absence of immunoglobulins. This condition is most commonly a result of a genetic mutation within the BTK gene, which is part of a signal cascade system critical for the maturation and production of B cells within the bone marrow [1,2]. The most common genetic variation is the X-linked genetic mutation because the BTK gene is found on the long arm of the X chromosome [1]. Without the necessary antibody-mediated defense mechanism, the body becomes susceptible to various and potentially life-threatening bacterial infections. Typically, patients with agammaglobulinemia become susceptible to bacterial infections after the maternal antibodies shared within colostrum and breast milk wane around six to 12 months of age. With the lack of B cell production, the downstream consequence is the loss of plasma cells, which are the cells responsible for generating immunoglobulins, leading to the absence of humoral immunity. Typically, the diagnosis is made during the first year(s) of life due to recurrent sinopulmonary infections (underdeveloped tonsils and adenoids) and gastrointestinal infections (underdeveloped Peyer’s patches) that result in severe infections during childhood. Often, the most noticeable clinical finding outside of a severe bacterial infection is the absence of tonsils, adenoids, or lymph nodes during periods of infection. The diagnosis is supported by the detection of low immunoglobulin levels, approximately two standard deviations below the average level, and the absence of B cells (<1% of lymphocytes that are CD19+) as determined by flow cytometry [3]. Patients are often started on immunoglobulin replacement therapy, leading to notable improvements in outcomes compared to those who do not undergo treatment. The potential underlying causes of acute pericardial effusion are numerous and include viral, bacterial, malignancy, acute traumatic injury, and autoimmune disease. The overwhelming majority are idiopathic and of presumed viral etiology [4]. The most concerning consequence of pericardial effusion is the development of life-threatening cardiac tamponade, in which increasing intra-pericardial pressure overcomes intraventricular end-diastolic pressures, ultimately reducing venous return and leading to obstructive shock [5]. Consequently, the prompt identification of tamponade physiology and rapid treatment is imperative, independent of the identification of the specific underlying etiology. Patients with pericardial effusion may present with nonspecific symptoms, including chest pain, dyspnea, and fatigue. Patients may have preceding or concomitant viral symptoms. Because the pericardium has the potential to stretch, large-volume effusions (greater than 1000 mL) that develop gradually may not cause tamponade physiology, while significantly smaller effusions (less than 250 mL) that develop rapidly may result in acute decompensation [6]. Physical signs indicative of cardiac tamponade encompass a spectrum of manifestations, such as tachycardia, pulsus paradoxus, and Beck’s triad, characterized by jugular venous distention, muffled heart sounds upon auscultation, and hypotension. Electrocardiographic findings of diffuse ST elevations suggest pericarditis, as seen in our patient’s case, while decreased QRS amplitude or electrical alternans suggests effusion, but are not diagnostic of tamponade. POCUS provides the advantage of allowing timely, direct visualization and estimation of effusion volume while assessing its consequent impact on cardiac dynamics. A pericardial effusion may be visualized in any of the four standard cardiac windows, manifesting as an anechoic (black) fluid collection between the pericardium and myocardium. Findings diagnostic of cardiac tamponade include right atrial collapse during ventricular systole, IVC plethora, and right ventricular collapse during ventricular diastole [7]. The diastolic collapse of the right ventricle is roughly 90% sensitive and 100% specific for cardiac tamponade [8]. These physiologic changes result in impaired RV filling and subsequent obstructive shock as downstream left ventricle (LV) filling is reduced. Additionally, advanced POCUS methods that focus on capturing flow velocity variations across the mitral and tricuspid valves represent sonographic evidence of pulsus paradoxus. In normal physiology, velocity across the mitral valve inflow region typically varies by 15% or less with respirations, and the tricuspid valve inflow variation is typically 25% or less with respirations. If the velocity variations increase (mitral valve to >25% and tricuspid valve to >35%), that suggests sonographic evidence of pulsus paradoxus [9]. Immediate, interim management for cardiac tamponade entails pericardiocentesis, a procedure that may be conducted at the bedside within the ED. However, for definitive intervention, more invasive measures such as pericardiotomy or even thoracotomy may be warranted, especially in cases involving cardiac arrest within the context of trauma and hemorrhagic pericardial effusion [7]. Purulent pericarditis, a rare infection affecting the pericardial space, occurs with an estimated incidence of approximately one in 18,000 individuals. It often occurs secondary to the spread of a neighboring infection. Purulent pericarditis is exceedingly rare in adult patients with XLA, with documented occurrences limited to just three other reported cases [10]. The disease presents as an acute, rapidly progressing febrile illness. While only half of patients exhibit classic manifestations of acute pericarditis - such as chest pain, pericardial friction rub, and pulsus paradoxus - fever is virtually ubiquitous in all cases. Diagnosis requires pericardiocentesis, where the fluid is subjected to macroscopic examination, revealing exudative characteristics. After draining the pericardial space, additional treatment is required with IV antibiotic therapy. Notably, the mortality rate associated with purulent pericarditis approaches nearly 100% in the absence of prompt intervention. Even with treatment, mortality rates remain alarmingly high, reaching approximately 40% [10]. Hence, timely recognition of this condition is imperative. Additionally, it is pertinent to note that immunosuppression serves as a recognized risk factor, further complicating the prognosis.

## Conclusions

POCUS stands as an indispensable skill essential for all acute care physicians. The versatility of ultrasound shines in its ability to quickly diagnose and initiate prompt treatment for pericardial tamponade, irrespective of its underlying cause. In this case, the patient was diagnosed with untreated agammaglobulinemia, a seldom-seen immunodeficiency disorder, which subsequently precipitated tamponade due to purulent pericarditis. Purulent pericarditis, albeit rare, poses a significant threat with its high mortality rate, often stemming from the contiguous spread of infection. Long-term management of patients with agammaglobulinemia necessitates immunoglobulin therapy to mitigate the risk of life-threatening infections, underscoring the importance of longitudinal outpatient care in addressing their unique medical needs.
